# The impact of flow-induced forces on the morphogenesis of the outflow tract

**DOI:** 10.3389/fphys.2014.00225

**Published:** 2014-06-17

**Authors:** Stefanie V. Biechler, Lorain Junor, Ashlie N. Evans, John F. Eberth, Robert L. Price, Jay D. Potts, Michael J. Yost, Richard L. Goodwin

**Affiliations:** ^1^Department of Cell Biology and Anatomy, School of Medicine, University of South CarolinaColumbia, SC, USA; ^2^Instrumentation Resource Facility, School of Medicine, University of South CarolinaColumbia, SC, USA; ^3^Department of Surgery, Medical University of South CarolinaColumbia, SC, USA

**Keywords:** outflow tract, bioreactor, mechanotransduction, fibrotic development, hemodynamics

## Abstract

One percent of infants are born with congenital heart disease (CHD), which commonly involves outflow tract (OFT) defects. These infants often require complex surgeries, which are associated with long term adverse remodeling effects, and receive replacement valves with limited strength, biocompatibility, and growth capability. To address these problematic issues, researchers have carried out investigations in valve development and valve mechanics. A longstanding hypothesis is that flow-induced forces regulate fibrous valve development, however, the specific mechanisms behind this mechanotransduction remain unclear. The purpose of this study was to implement an *in vitro* system of outflow tract development to test the response of embryonic OFT tissues to fluid flow. A dynamic, three-dimensional bioreactor system was used to culture embryonic OFT tissue under different levels of flow as well as the absence of flow. In the absence of flow, OFT tissues took on a more primitive phenotype that is characteristic of early OFT cushion development where widely dispersed mesenchymal cells are surrounded by a sparse, disorganized extracellular matrix (ECM). Whereas OFT tissues subjected to physiologically matched flow formed compact mounds of cells, initated, fibrous ECM development, while prolonged supraphysiological flow resulted in abnormal tissue remodeling. This study indicates that both the timing and magnitude of flow alter cellular processes that determine if OFT precursor tissue undergoes normal or pathological development. Specifically, these experiments showed that flow-generated forces regulate the deposition and localization of fibrous ECM proteins, indicating that mechanosensitive signaling pathways are capable of driving pathological OFT development if flows are not ideal.

## Introduction

Outflow valve defects are involved in the most common Congenital Heart Disease (CHDs) (Schoen, 2008). Because valve defects are associated with stenosis and regurgitation may not become problematic until later in life, they are among the most costly and clinically relevant CHDs. For this reason, valve replacements, the second most common cardiac surgery (Combs and Yutzey, 2009), are being investigated to develop better therapies and treatments for the large population of both children and adults affected by valve disease. It is imperative that a portion of this research is focused on progressing a greater understanding of the developmental mechanisms that regulate healthy valve morphogenesis.

It is well established that atrioventricular cushions (AVCs) and outflow cushions (OFCs) follow similar developmental mechanisms early in development (Hinton and Yutzey, 2011). In both cases, the valves are derived from the endocardial cushion tissue; however, OFCs lag slightly behind the AVCs and have a cellular contribution from neural crest cells (NCCs) (Armstrong and Bischoff, 2004; Hinton and Yutzey, 2011). In chick embryos, these NCCs undergo epithelial to mesenchymal transformation (EMT) around HH stage 11 before they begin migration into the dorsal OFT (Kirby and Hutson, 2010). By HH stage 21, these migrating NCCs contribute to the OFCs (Webb et al., 2003) and follow similar signaling mechanisms as those found in AVCs, such as BMP signaling. Much like in the AVCs, there is evidence that flow-responsive signaling cascades play a role in the early development of OFCs. The misregulation of signaling pathways, such as KLF2/TGFβ and RhoA/ROCK, lead to diseased semilunar valves that are characterized by increased, disorganized extracellular matrix (ECM) (Hinton et al., 2006). Further, studies have shown that reduced flow in the OFT results in impaired valve formation and septation (Hove et al., 2003), indicating that the connection between flow and development via specific signaling mechanisms is crucial to normal valve development.

In a previous study, the impact of flow-induced forces on AVC development was investigated using an *in vitro* system of valve development with an extruded collagen tube scaffold (Tan et al., 2012). Due to relatively large diameter of the original tube scaffold (4 mm), low velocity laminar flow profiles were required to prevent the explant from washing away, restricting the range of potential flow magnitudes. For this reason, a new, molded (cast) collagen scaffold that contained a 0.65 mm restriction was used to achieve physiological levels of flow in an *in vitro* system of OFT development. In the study presented here, OFT explants were exposed to physiological and pathological levels of flow to investigate the impact of flow-induced forces on the OFT cushion of the heart. Ultimately, this study contributes to the ever-increasing research efforts that seek to elucidate the developmental mechanisms that regulate healthy vs. pathological semilunar (SL) valve morphogenesis.

## Materials and methods

### Culture of OFT explants in the fluid flow bioreactor system

**Figure 1:**
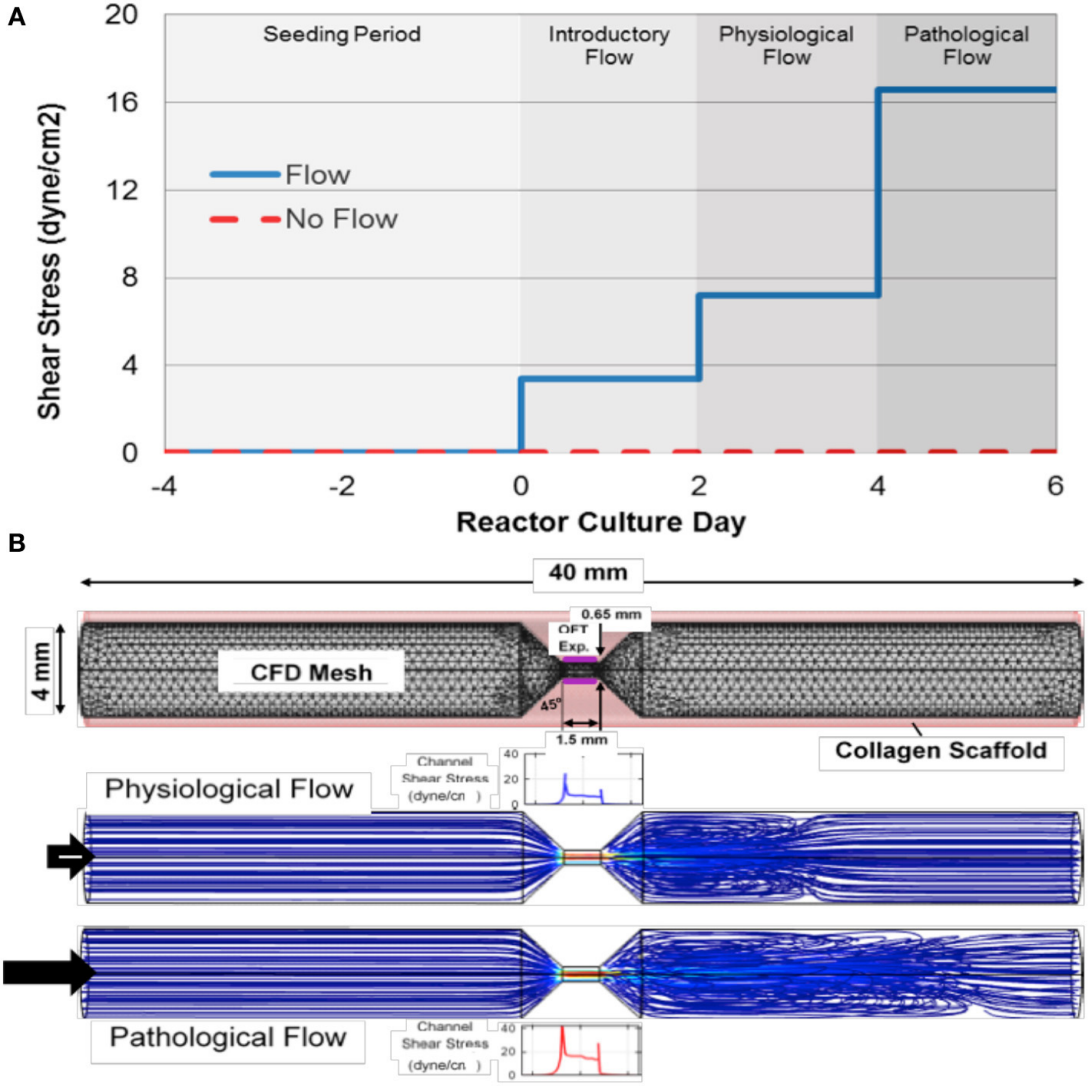
**Experimental design for flow-cultured outflow tract explants in molded scaffolds. (A)** The total outflow tract (OFT) explant culture period (from time of dissection) was 8 days in the physiological case and 10 days in the pathological case. After the 4 day seeding period, a 2 day introductory flow period was used before flow was ramped to sub-physiological. After the 2 day introductory period, some flow groups and no flow controls were removed and some remained on for an additional 2 days under physiological levels of flow. These final flow groups, and no flow controls, were then removed. **(B)** To determine the flow needed to achieve physiological or pathological levels wall shear stress at the center of the channel for outflow tract (OFT) explants (Purple), a 3-dimensional computational fluid dynamics (CFD) model was generated with precise geometry. Note the high fluid velocity in the seeding channel (red streamlines) and eddy formation downstream of the channel.

Avian embryos that have not yet reached hatching stage are not considered live animals. Nonetheless AVMA Guidelines for Euthanasia protocols were followed in carrying out these experiments. HH stage 25 OFT explants (approximately 10 per tube) were isolated by cutting the OFT at the heart/OFT boundary and then the OFT/aortic sac boundary. These tissues contain both conotruncal and intercalated cushions that participate in aorticopulmonary septation and OFT valve formation. The OFT explants were then butterflied open and seeded onto the narrow channel section of the flow channel surface of the molded “hourglass”-like Col1 scaffold (Figure 1). HH stage 25 OFT tissues were selected because NCCs have already migrated to the OFCs, and all cell types necessary for cushion development are present. The explants were allowed to attach to the scaffolds during a 4 day static culture period. The scaffolds containing OFT explants were then sutured onto the glass nozzles of the bioreactor. Reactors contained culture media: Dulbecco's Modified Eagle Medium (#50-013-PB, Corning Cellgro, Mediatech, Inc.) and 10%- Fetal Bovine Serum (FBS) (#F-0500-A, Atlas Biologicals, Inc.) with Penicillin Streptomycin (#P7794 and #S6501, Sigma-Aldrich), Amphotericin B (#A9528, Sigma-Aldrich), and Minocycline (#M9511, Sigma-Aldrich). A peristaltic pump (Ismatec Ecoline VC-MS/CA8-6, IDEX Health & Science) used with 1.85 mm inner diameter cassette tubing, was connected to the tubing circuit in the case of flow to generate physiological or pathologically high levels of flow. “No-flow” control tubes were also sutured into bioreactors that were connected to a closed loop of stagnant media (not connected to the pump).

AVCs and OFCs have similar dimensions and are exposed to similar blood flow rates early on as they follow common developmental processes (Hinton et al., 2006; Liu et al., 2011). It is therefore likely that the level of shear stress is comparable between the two cushion types at early stages of development. A mean shear stress of 7.2 dyne/cm^2^ was selected as the desired physiological condition based on previous studies of developing AVCs (Liu et al., 2009, 2011; Yalcin et al., 2011). To model the development of outflow tract stenosis, we chose a mean shear stress of 16.6 dyne/cm^2^ as a pathological flow condition. Since local flow profiles influence explant morphology we characterized the flow throughout the cast collagen tube by creating a three-dimensional computation fluid dynamics (CFD) model of the intraluminal culture media. The CFD model was then used to determine the flow rate needed to reach either 7.2 or 16.6 dyne/cm^2^ in the seeding channel of the scaffold.

Poiseuille's equation provided an analytical starting point for the CFD model; however, it was not sufficient to fully characterize the flow due to channel entrance length restrictions. The CFD model was implemented using COMSOL Multiphysics 4.2a (Burlington, MA) to represent the molded collagen scaffold. The geometry was defined to match the dimensions of the mold's inner pins (scaffold lumen) and the structure was discretized (Figure 1B). A rigid wall assumption was applied and the media solution was considered Newtonian and incompressible with properties similar to that of water at 37 degrees C, (density, ρ, of 993.4 kg/m^3^ and dynamic viscosity, μ, of 0.692 cP). The no-slip boundary condition was invoked at the wall and a pressure of 0 dyne/cm^2^ was applied at the outlet. For the physiological condition, the inlet flow was adjusted until there was a mean shear stress of 7.2 dyne/cm^2^ (flow rate of 24.3 mm^3^/s) or in the case of the pathological flow; a mean shear stress of 16.6 dyne/cm^2^ (flow rate of 48.7 mm^3^/s) was met at the center of the seeding channel. The Reynolds number at pathological conditions was found to be 137 indicating that the flow in this region was laminar for all flow conditions.

The culture protocol used in these experiments is illustrated in Figure 1A. After the static 4-day seeding period, tubes were sutured onto bioreactors and the flow groups were exposed to introductory shear stresses of 3.4 dyne/cm^2^ (flow rate of 12.2 mm^3^/s) for 2 days as this initial rate was found be permissive for newly attached cushion tissues in previous experiments (Tan et al., 2012). Flow was then ramped up to match the mean physiological shear stress of 7.2 dyne/cm^2^ (flow rate of 24.3 mm^3^/s) for 2 days. To model the development of OFT stenosis, cultures were on reactors for an additional 2 during which the mean shear stress was increased to 16.6 dyne/cm^2^ (flow rate of 48.7 mm^3^/s). These pathological flow samples and 10 day no flow control cultures were then removed from reactors to represent the pathological case.

### 3D reconstruction of scaffold with OFT explants

**Figure 2:**
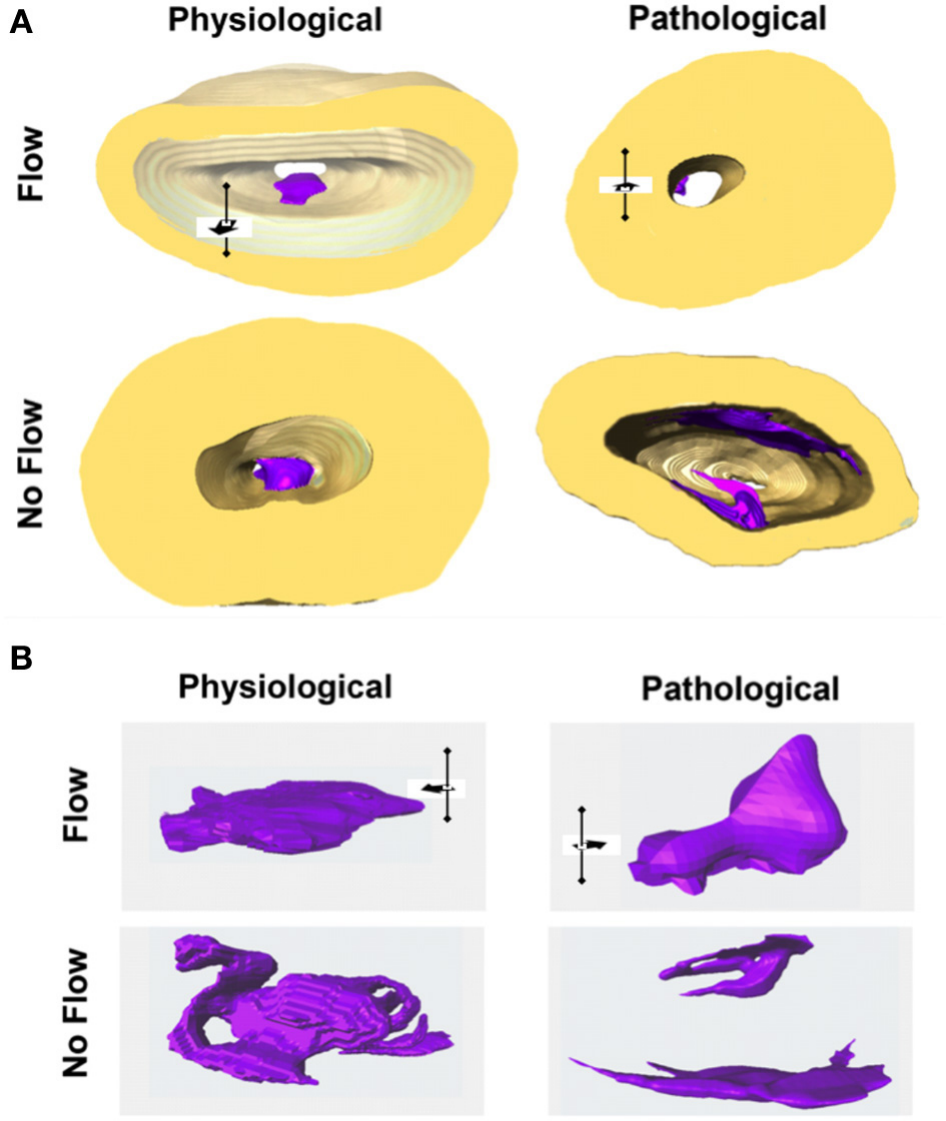
**Flow affects outflow tract explant morphology. (A)** All outflow tract (OFT) sample groups were three-dimensionally reconstructed with labeled scaffold (gold) and explant tissue (purple). **(B)** Physiological flow groups exhibited a mound shape while pathological flow groups exhibited a smaller, leaflet morphology. In the case of no flow, tissue formed a loose network and occluded the lumen of the flow channel (comparisons are not to scale). Flow direction is indicated by the black arrow in three dimensional space.

Cultured scaffolds with explants from all four groups (physiological flow with no flow control and pathological flow with no flow control, *n* = 2 for each group) were reconstruction three-dimensionally so that tissue morphogenesis in response to flow could be investigated. The samples were fixed in 4% PFA (in PBS buffer pH = 6.8) overnight, embedded in 5% agarose, and sectioned in 200 μm increments using a vibratome (Oxford). Serial sections were mounted on slides, stained with HE, and imaged using a Zeiss Stemi DV4 Stereomicroscope (Carl Zeiss, Inc.). These serial images were then used to three-dimensionally reconstruct the tubes with explants in AMIRA (Visage Imaging, Inc.). After serial section alignment, the scaffold and explant tissue were labeled as separate materials (Figure 2, gold and purple, respectively).

### Quantification techniques

To quantify the effects of flow vs. no flow on the OFT explants, qRT-PCR, confocal microscopy and RhoA activation ELISAs were performed. Real-time PCR primers were designed in Primer3 (SimGene) and are listed in Table 1. The relative expression of genes of interest (GOI) was calculated [relative to an acidic ribosomal binding protein (ARBP) standard] using the Pfaffl method, which normalizes the differences between the reaction kinetics of different primer sets (Pfaffl, 2001). In addition to qRT-PCR runs on the *in vitro* samples (*n* = 3 per group; 4 groups), transcript levels were also quantified using freshly dissected OFTs from HH stages 25, 28, and 30 (*n* = 3 per group). These represent the *in vivo* stages modeled over the time course of the *in vitro* experiments.

**Table 1:** **Chick OFT experiment primer sequences**.

**qRT-PCR GOI**	**Forward 5'-to-3'**	**Reverse 5'-to-3'**
ARBP	CCT GTG ATG TGA CTG TGC	ACT TTG TCT CCG GTC TTA ATC
Tenascin C	AGG ACA CAG CCT CTG CAA GT	TAC TGC CCC TGA GAG CTG AT
Periostin	GGA TGG TAT GAG AGG ATG TC	GCA AAG AAA GTG AAT GAA CC
Elastin	GCG CAC AAG GGA AAT ACT GT	CTC AGC TTG GTG GGG ATT TA
Col1α1	TGC TGT TGA TAG CAG CGA CT	GTG TCC TCG CAG ATC ACC TC
Col6α1	TCC AGA TTG CCA AGG ATT TC	AGC AGG TTT TCC TTG CTG AA
RhoA	CAG CAC CCT GCA CTT GAG TA	GCA TCC TGT GAG TGC AGA AA
Rac	CCT TTG AAA ATG TCC GTG CT	CCT CGC TGT GTA AGT GCT GA
FAK	ATT GCT GCT AGG AAC GTG CT	ACC GTC GGA AGT TGA TTG AC
Vinculin	ATG AAG CTG GAA AAG CAG GA	TTT GCA GTG AGC AAA TCC AG
Paxillin	GCC CTA AAT GGC ACT GTG AT	CTT CTG CTT GTT TGG GAA GC
KLF2	GCT TCT ACC AGA CAA ACC CG	CAG GAC TGG CCC ATA ACT GT
TGFβ1	GCT CTG TAC AAC AGC ACC CA	ACG AAG AAG ATG CTG TGG CT
TGFβ2	GAG AAA GCC AAC CAC AGA GC	GGT ACA GCT CTA TCC GCT GC
TGFβ3	TAA ATC CTC TCG TTG TGG GG	CAC AAT GAG TTG GGC ATT TG
CTGF	CAG GTT TAG CGA CAG CAA CA	GTA ACC TAA CTG CCG CAA GC
αSMA	GGG GAT GAT GCT CCA AGA GCA GTT T	CCT CAG GTG CAA CAC GGA GCT CAT T
α-Actinin	CAC TTG CGG CAG TAT GAG AA	CAG GGG TCA GAA TCT GGT TT
Vimentin	CCG ACA GGA TGT TGA CAA TG	TCT TAG CAG CAA CGC TTT CA

Scaffolds were processed, sectioned, and prepared for confocal microscopy. Sections were stained for tenascin C (#AB19013, Millipore), periostin (#AB1404.1, Abcam), elastin (#AB21610, Abcam), Col6 (#5C6, Developmental Studies Hybridoma Bank), F-actin (rhoadamine phalloidin, R415, Invitrogen), fibronectin (#IST-9, Abcam), and P-Histone H3 (#9713S, Cell Signaling Technology, Inc., Boston, MA) using a concentration of 1:200 in BSA/PBS overnight at 4°C. “No primary antibody” controls were incubated in PBS only overnight at 4°C. The sections were then stained with Alexa-conjugated secondary antibodies (Invitrogen) and Dapi (#D21490, Invitrogen) overnight at concentrations of 1:200 and 1:5000, respectively, overnight at 4°C. Sections were mounted and imaged using a Zeiss LSM 510 META confocal laser scanning microscope (Carl Zeiss). Images were collected using identical microscope settings per antibody so that relative expression could be determined between groups. Sample numbers per group are listed in Table 2 for each antibody. Z-stacks were collected for each sample group using a Nyquist sampling approach and imported into the AMIRA software package. Once in AMIRA, the image stacks were rendered using identical threshold values for each channel across all samples. The module that yields voxels for each channel was then used to generate volumes for each protein of interest. This volume was taken as a ratio to the Dapi volume, which were to compare across all sample groups. In this way, the volume of protein is normalized by the number of nuclei in the tissue.

**Table 2:** **Sample number per group in OFT experiment confocal analysis**.

**Antibody**	**Physiological flow**	**Phys. no flow control**	**Pathological flow**	**Path. no flow control**
Tenascin C	*n* = 4	*n* = 5	*n* = 5	*n* = 4
Periostin	*n* = 7	*n* = 8	*n* = 8	*n* = 6
Elastin	*n* = 8	*n* = 8	*n* = 4	*n* = 6
Col6	*n* = 3	*n* = 3	*n* = 3	*n* = 3
F-Actin	*n* = 3	*n* = 5	*n* = 7	*n* = 5
Fibronectin	*n* = 8	*n* = 10	*n* = 6	*n* = 10
P-Histone H3	*n* = 9	*n* = 3	*n* = 6	*n* = 5

RhoA activity was quantified as described previously (Tan et al., 2012) using colorimetric ELISA assays (Total RhoA ELISA Kit, #BK150, Cytoskeleton Inc.; RhoA Activation Assay Biochem Kit, #BK124, Cytoskeleton Inc.). The percentage of active RhoA was subsequently determined using the dividend of active and total RhoA for each sample (physiological flow, pathological flow, and pathological no flow control, *n* = 3; physiological no flow control, *n* = 4). Activation assays were also performed on freshly dissected OFCs from HH stages 25, 28, and 30 (*n* = 3 per stage) to quantify *in vivo* activation levels.

### Transmission electron microscopy

Transmission Electron Microscopy (TEM) was performed to investigate the ultrastructure of the explants (*n* = 1 per group) were fixed in 2.5% glutaraldehyde in PBS (pH = 7.2) overnight at room temperature. Samples were subsequently washed in PBS, post-fixed in a 1% aqueous osmium tetroxide solution, and dehydrated in a graded ethanol series. The samples were then embedded in PolyBed 812 (Polysciences, Inc., Warrington, PA) and sectioned at 100 nm. Sample images were gathered with a JEOL 200CX TEM (JEOL USA, Inc., Peabody, MA).

### Statistical analysis

All data were expressed as Mean ± s.e.m. Statistical comparisons were performed using ANOVA or independent *t*-test by Prism 6 (GraphPad Software, Inc., La Jolla, CA). *P* values <0.05 (^*^) were considered to be statistically significant.

## Results

### Flow-induced forces affect OFT explant morphogenesis

The flow impacted the overall geometry of the OFT explant tissue (Figure 2). In the case of physiological flow, the explants formed a compact mound at the inflow side of the seeding channel. Explants exposed to pathologically high levels of flow and two additional days of culture formed a small, leaflet-like structure extending in the direction of flow from the seeding channel inlet and extending downstream through the channel of the scaffold. These pathological flow samples all had small areas of explant/scaffold interface. In no flow control groups, explants formed a loose, spider web-like network that bridged the entire luminal diameter of the scaffold channel.

### The effects of flow on OFT explant fibrous ECM proteins

**Figure 3:**
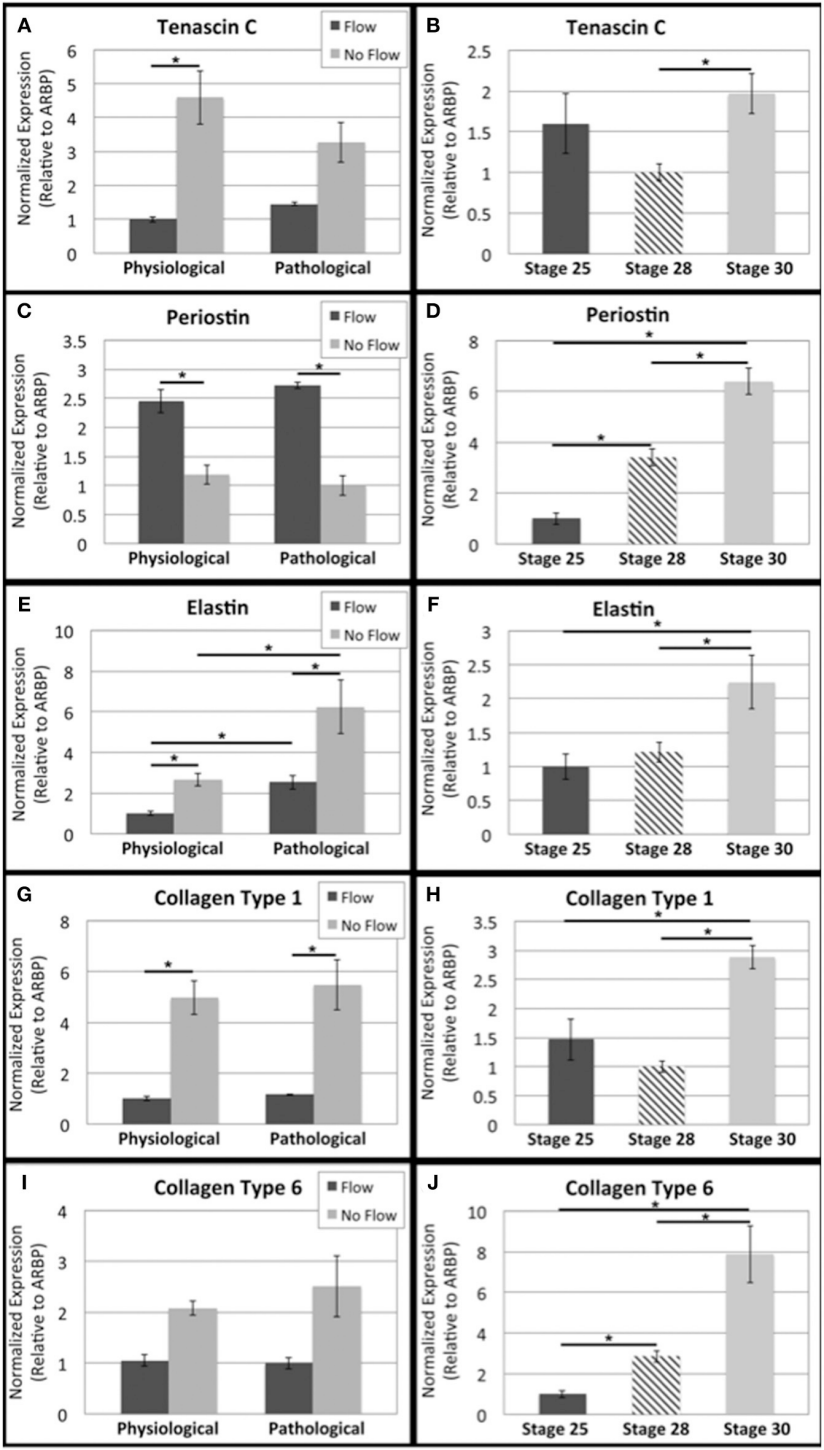
**Flow affects fibrous extracellular matrix protein transcript levels in outflow tract**. Quantitative Real Time PCR was performed on the four culture groups and on freshly dissected outflow cushions (OFCs) at three stages in development (HH stage 25, 28, and 30). **(A,B)** Tenascin C transcript level was higher in the absence of flow and increases between HH stage 28 and 30. **(C,D)** Periostin transcript level was higher in the presence of flow and appears to steadily increase from HH stage 25 to 30. **(E,F)** Elastin transcript level was higher in the absence of flow and in pathological groups and transcript level appears to increase between HH stage 28 and 30. **(G,H)** Type 1 collagen (Col1) transcript level was higher in the absence of flow with no difference between physiological and pathological groups. Col1 transcript appears to increase at HH stage 30. **(I,J)** Type 6 collagen (Col6) followed a similar trend to Col1 but with no statistical significance and Col6 transcript levels appear to steady increase from HH stage 25 to 30. (^*^P < 0.05).

To investigate changes in transcript levels of fibrous ECM genes that are associated with cellular responses to mechanical stimuli, qRT-PCR was performed (Figure 3). Tenascin C, elastin, Col1, and Col6 transcripts were downregulated in flow cases compared to their no flow controls (Figures 3A,E,G,I, respectively), while periostin transcript was upregulated in response to flow (Figure 3C). Elastin, the only transcript that showed significant differences between physiological and pathological groups, was upregulated in the extended culture pathological groups (flow and no flow control) (Figure 3E). To enable the comparison of these *in vitro* results to changes transcript levels that occur during relevant stages of heart, qRT-PCR for freshly dissected OFCs from HH stage 25, 28, and 30 embryos. *In vivo*, all fibrous ECM protein transcripts appear to be upregulated between HH stage 28 and 30 (Figures 3B,D,F,H,J). While transcriptional changes were seen in response to flow, to assess the changes in protein deposition, confocal microscopy was also performed.

**Figure 4:**
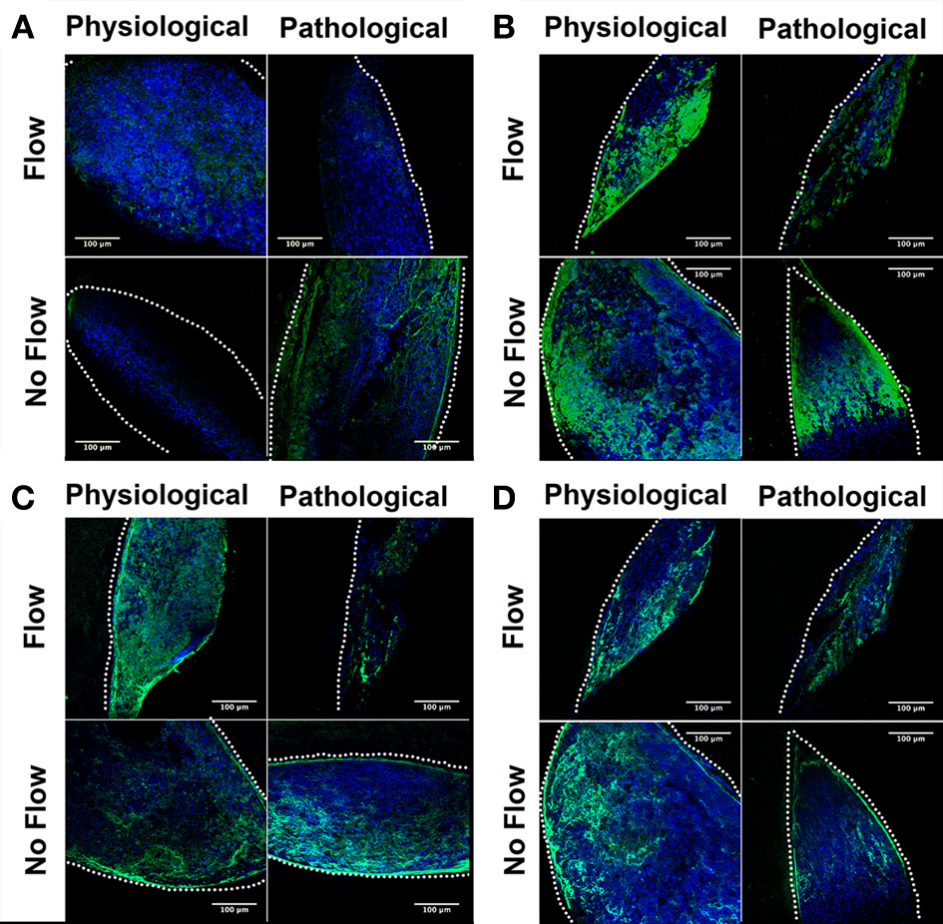
**Flow affects fibrous extracellular matrix protein localization in outflow tract**. Outflow tract (OFT) explant samples were stained with Dapi (blue) or for several hallmark extracellular matrix (ECM) proteins (green): **(A)** Tenascin C **(B)** Periostin **(C)** Elastin **(D)** Type 6 Collagen (Col6). In all no flow controls, ECM staining was throughout the tissue network, with a greater staining concentration at the scaffold interface. In each case of flow, tenascin C, periostin, and Col6 were localized toward the inlet side of the cushion **(A,B,D)** while elastin staining presented throughout the cushions with greater concentration on the outlet side. Scale bars are all 100 μm. The dotted lines indicate the scaffold wall.

Confocal microscopic analysis indicated that both physiological and pathological flow promoted organized localization of several hallmark fibrous ECM proteins. Tenascin C and Col6 (Figures 4A,D, respectively) were localized on the inflow (upstream) side of the OFT cushions. Periostin (Figure 4B) was diffuse in the flow-cultured tissue with increased staining intensity along the luminal edge. Elastin (Figure 4C) staining was diffuse in the flow-cultured cushions with robust deposition on the outflow (downstream) side. All no flow control groups showed ECM staining of all four fibrous proteins throughout the tissue, with greater staining intensity at the scaffold-tissue interface, where some cells invaded the scaffold. This intrascaffold cell localization was not observed in any of the flow-cultured explants. Images were taken using the same microscope settings so that the mean fluorescence intensity (green and blue channels) could be determine and relative deposition quantified.

**Figure 5:**
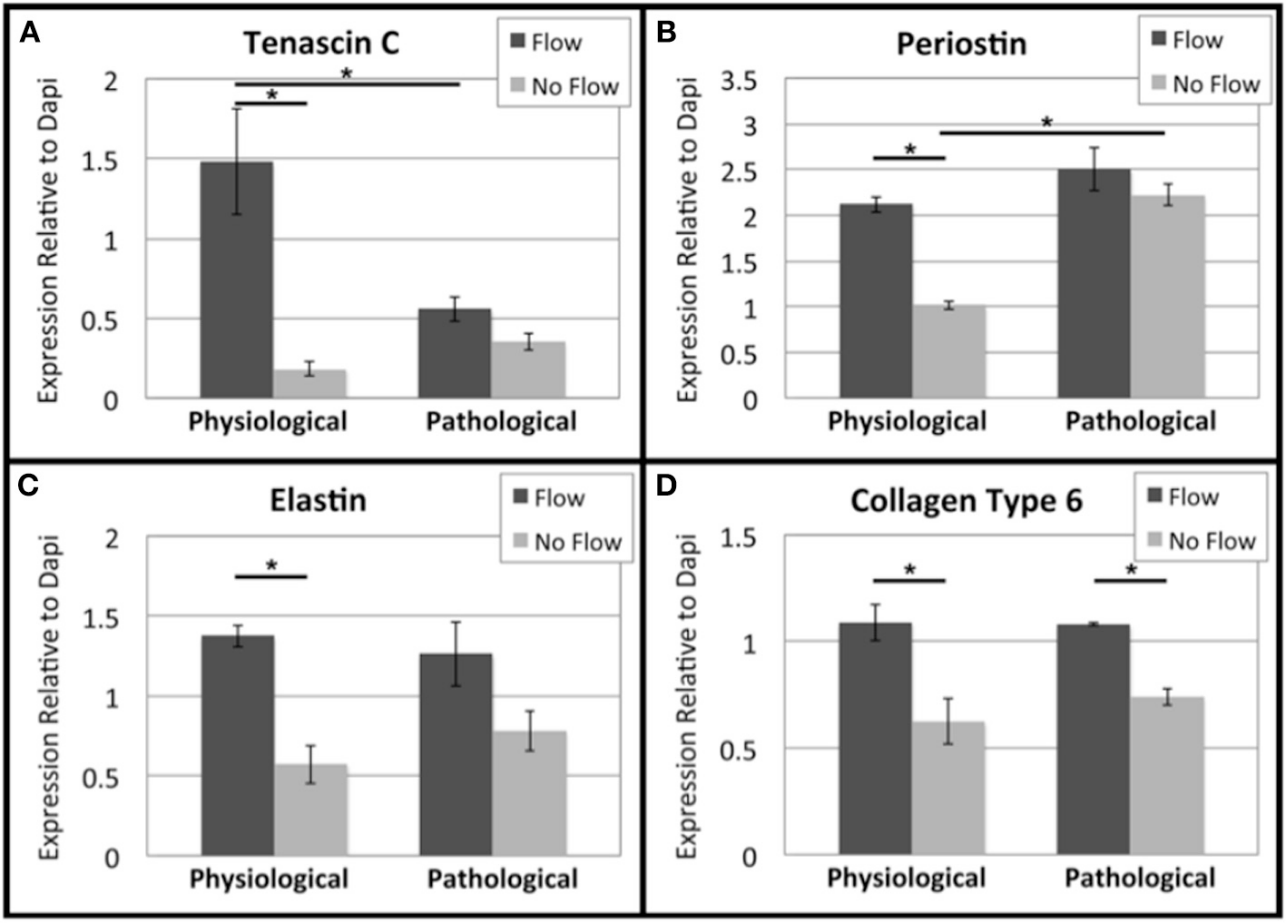
**Flow affects fibrous extracellular matrix protein expression in outflow tract**. Confocal microscopy was performed for outflow tract (OFT) explants at the same settings and the mean fluorescence intensity of each fibrous extracellular matrix (ECM) protein was analyzed relative to Dapi staining. Expression quantifications were averaged for each of the four culture groups. **(A)** Tenascin C was upregulated under physiological flow compared to the no flow control and pathological flow. **(B)** Periostin was upregulated under physiological flow compared to the no flow control. The extended no flow culture period also appeared to upregulate periostin. **(C)** Elastin was upregulated under physiological flow compared to the no flow control and no differences were seen between physiological and pathological groups. **(D)** Col6 followed a similar trend to elastin. (^*^P < 0.05).

Confocal image analysis indicated that flow, physiological or pathological, resulted in increased deposition of tenascin C, periostin, elastin, and Col6 when compared to no flow controls (Figure 5). Tenascin C levels were significantly higher in the physiological flow group than in the pathological flow group. Periostin deposition was significantly higher in the 8 day no flow group (pathological control) than in the 6 day no flow control. Elastin and Col6 exhibited similar expression patterns and neither ECM protein showed a change between the physiological and pathological groups.

### The effects of flow on cytoskeletal actin and fibronectin

**Figure 6:**
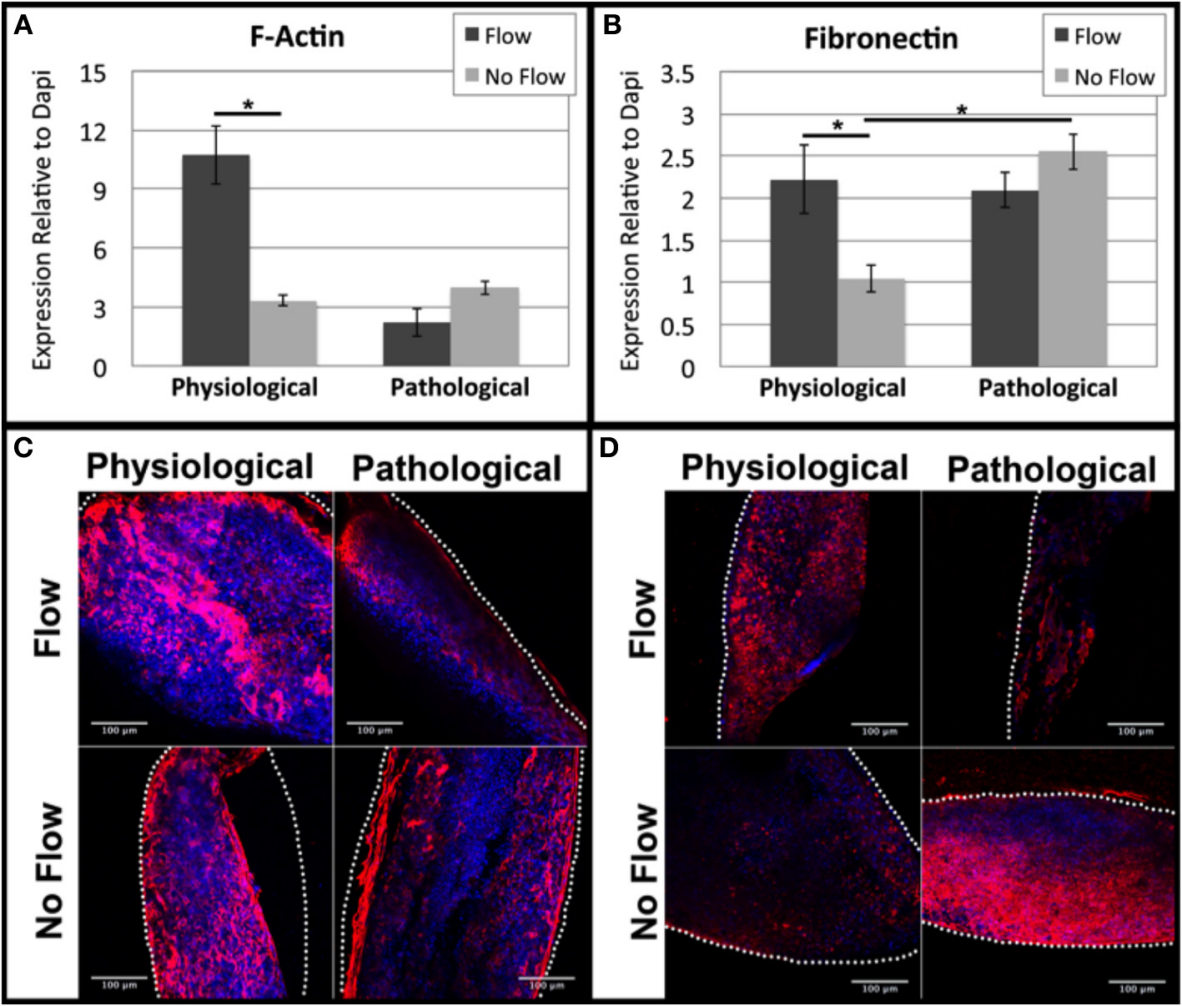
**Flow affects cytoskeletal actin and extracellular fibronectin in OFT cells**. Mean fluorescence intensity relative to Dapi was quantified from confocal imaging under the same setting for each outflow tract (OFT) explant culture group. **(A)** F-actin expression was upregulated under physiological flow compared to no flow controls. **(B)** Fibronectin expression was upregulated under physiological flow compared to no flow controls and extended no flow culture time appeared to upregulate expression. **(C)** F-Actin and **(D)** fibronectin expression both exhibited diffuse staining in all sample groups with increased concentration at the scaffold-tissue interface. Scale bars are all 100 μm. The dotted lines indicate the scaffold wall. (^*^P < 0.05).

While ECM proteins contribute to a cushion's mechanical properties, their organization does not become pronounced until later in development when the stratified layers of ECM within the valves becomes pronounced (Hinton and Yutzey, 2011). Thus, at early stages in cushion development, the actin cytoskeleton and fibronectin (which acts as a ligand for integrin receptors i.e., cell/ECM binding), are likely to play a significant role in tissue stiffness. Accordingly, we analyzed F-actin and fibronectin deposition using confocal analysis. Physiological levels of flow increased F-actin and fibronectin deposition compared to the physiological no flow controls while pathological flow decreased F-actin and fibronectin protein expression compared to the pathological no flow controls (Figures 6A,B). F-actin and fibronectin were diffuse throughout the tissue with higher staining intensity at the scaffold interface in all sample groups (Figures 6C,D).

### Changes OFT explant RhoA signaling

**Figure 7:**
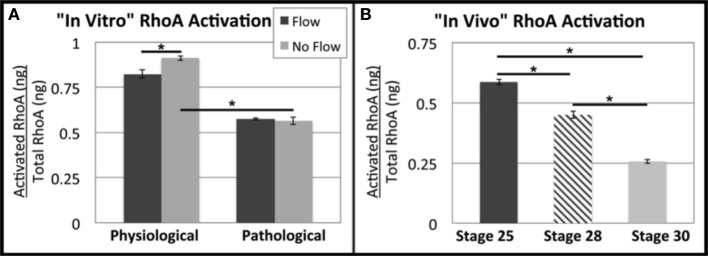
**Flow alters RhoA activation in outflow tract explants**. Total RhoA and active (GTP-bound) RhoA ELISAs were performed to quantify the ratio of active vs. total RhoA. **(A)** In the physiological group, more RhoA appeared to be activated in the absence of flow (no flow control), and RhoA activation was decreased in the pathological groups. **(B)** RhoA activity appears to decrease steadily from HH stage 25 to 30. (^*^P < 0.05).

Previously, we investigated RhoA as a possible mechanotransduction-signaling molecule in response to flow-induced forces. In these studies, we found that RhoA has a role in flow-induced ECM development in AVCs (Tan et al., 2012). Here, we found little to no difference in RhoA activation was seen between flow groups and their no flow control counterparts in OFT explants (Figure 7A). RhoA activation was higher in the physiological groups than the pathological groups. However, RhoA activity in freshly dissected OFTs was found to steadily decrease from HH stage 25 to 30 (Figure 7B).

**Figure 8:**
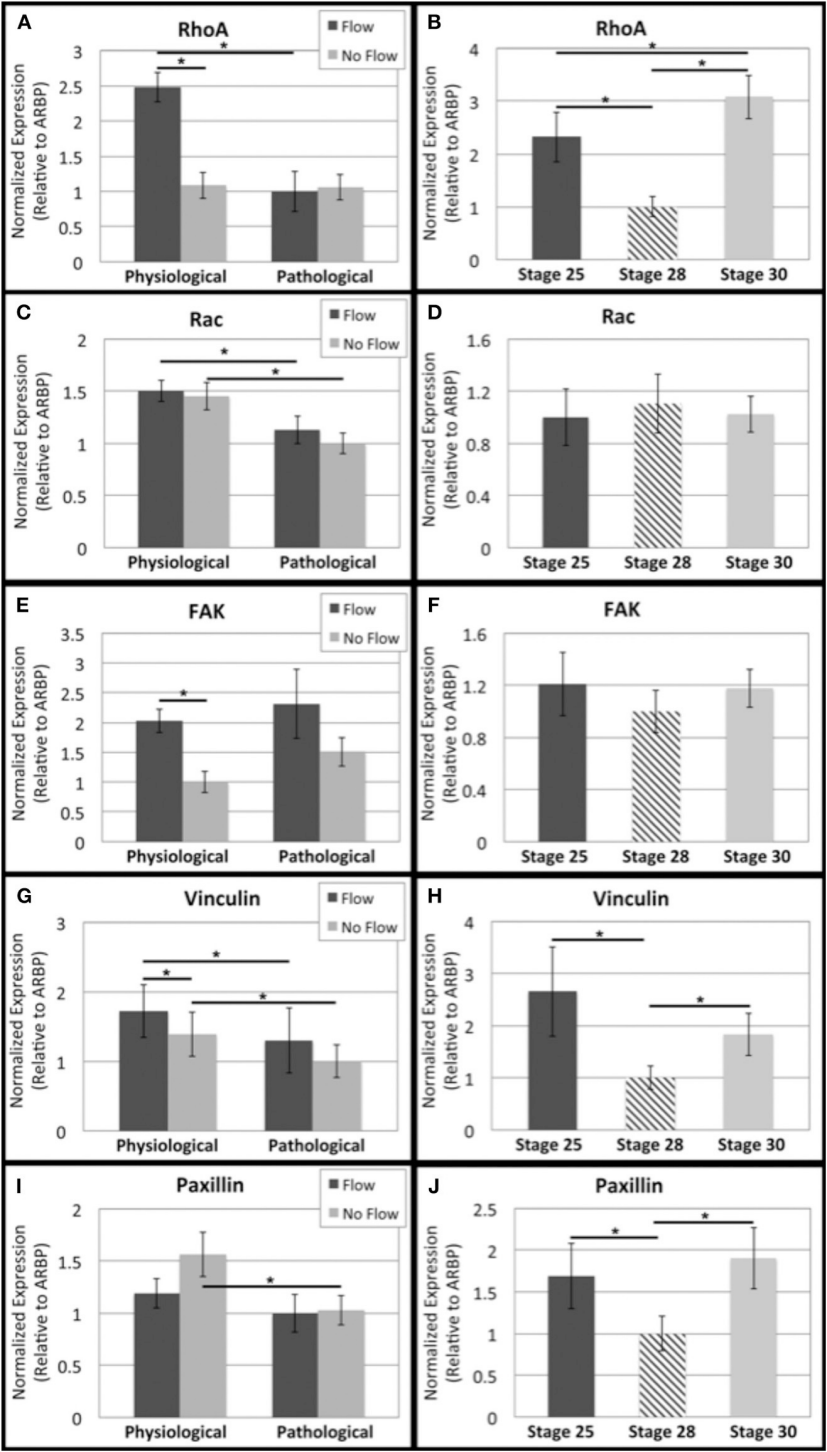
**Flow affects adhesion signaling transcript levels in outflow tract**. Quantitative Real Time PCR was performed on the four culture groups and on freshly dissected outflow cushions (OFCs) at three stages in development (HH stage 25, 28, and 30). **(A,B)** RhoA transcript level was higher under physiological levels of flow and appears to decrease from HH stage 25 to 28 and increase from HH stage 28 to 30. **(C,D)** Rac transcript level was higher in the physiological sample groups (flow and no flow control) and no significant differences were seen from HH stage 25 to 30. **(E,F)** FAK transcript level was higher in the case of flow and no significant differences were seen from HH stage 25 to 30. **(G,H)** Vinculin transcript level was higher for each flow case compared to no flow controls and transcript was greater in the physiological groups compared to the pathological groups. Transcript appears to, like RhoA, decrease from HH stage 25 to 28 and increase from HH stage 28 to 30. **(I,J)** Paxillin transcript level decreased with no flow culture time and appears to follow the same trend as RhoA and vinculin from HH stage 25 to 30. (^*^P < 0.05).

The small GTPases regulate actin reorganization and interact with a number of other pathways. So, the transcript levels of related genes were investigated (Figure 8). RhoA, FAK, and vinculin transcript levels were higher under physiological flow than the no flow controls (Figures 8A,E,G, respectively). Rac and vinculin transcript decreased with culture time in flow and no flow control groups (Figures 8C,G). The RhoA flow groups showed decreased transcript over time (Figure 8A) and the paxillin no flow control transcripts decreased with time (Figure 8I). No changes in FAK or Rac transcripts were seen from HH stage 25 to 28 (Figures 8D,F, respectively) while RhoA, vinculin, and paxillin all decreased from HH 25 to 28 and increased from HH 28 to 30 (Figures 8B,H,J, respectively).

**Figure 9:**
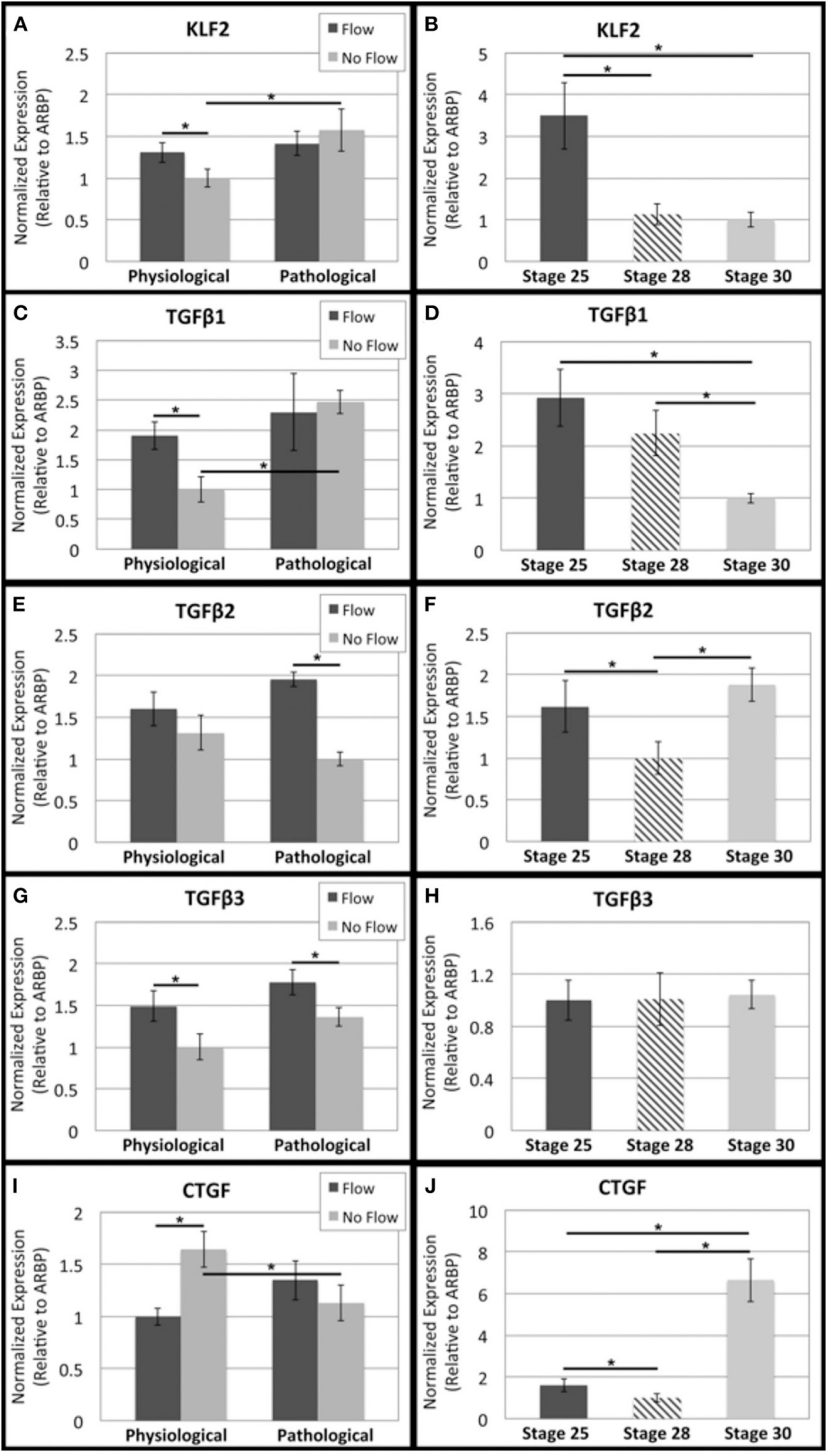
**Flow affects shear-responsive signaling transcript levels in outflow tract**. Quantitative Real Time PCR was performed on the four culture groups and on freshly dissected outflow cushions (OFCs) at three stages in development (HH stage 25, 28, and 30). **(A,B)** KLF2 transcript level was higher in the absence of flow and increased with increased static culture time. Transcript appears to decrease between HH stage 25 and 28. **(C,D)** TGFβ1 transcript level was higher in the presence of physiological flow compared to the no flow control. TGFβ1 transcript steadily decreases from HH stage 25 to 30. **(E,F)** TGFβ2 transcript level was higher in the presence of flow and appears to decrease from HH stage 25 to 28 and then increase from HH stage 28 and 30. **(G,H)** TGFβ3 transcript level was higher in the presence of flow with no difference between physiological and pathological groups. Transcript amount does not appear to change from HH stage 25 to 30. **(I,J)** CTGF transcript level was highest in the physiological no flow control group and appears to increase from HH stage 28 to 30. (^*^P < 0.05).

RhoA has also been connected to shear-responsive signaling molecules including KLF2 and the TGFβ proteins. A downstream target of RhoA in this pathway is CTGF, which is associated with fibrous ECM protein production. TGFβ 1, 2, and 3 transcript levels were all higher in the presences of flow (physiological and pathological) while KLF2 transcripts were increased in only physiological flow samples (Figures 9A,C,E,G, respectively). In the physiological groups, CTGF transcription was downregulated in response to flow (Figure 9I). *In vivo* OFTs were analyzed and showed that KLF2 and TGFβ1 transcripts decreased from HH stage 25 to 30 (Figures 6, 9 B,D, respectively) and CTGF increased from HH stage 25 to 30 (Figure 9J). No significant change in TGFβ2 and 3 transcripts were seen between HH stage 25 and 30 (Figures 9H,F, respectively).

**Figure 10:**
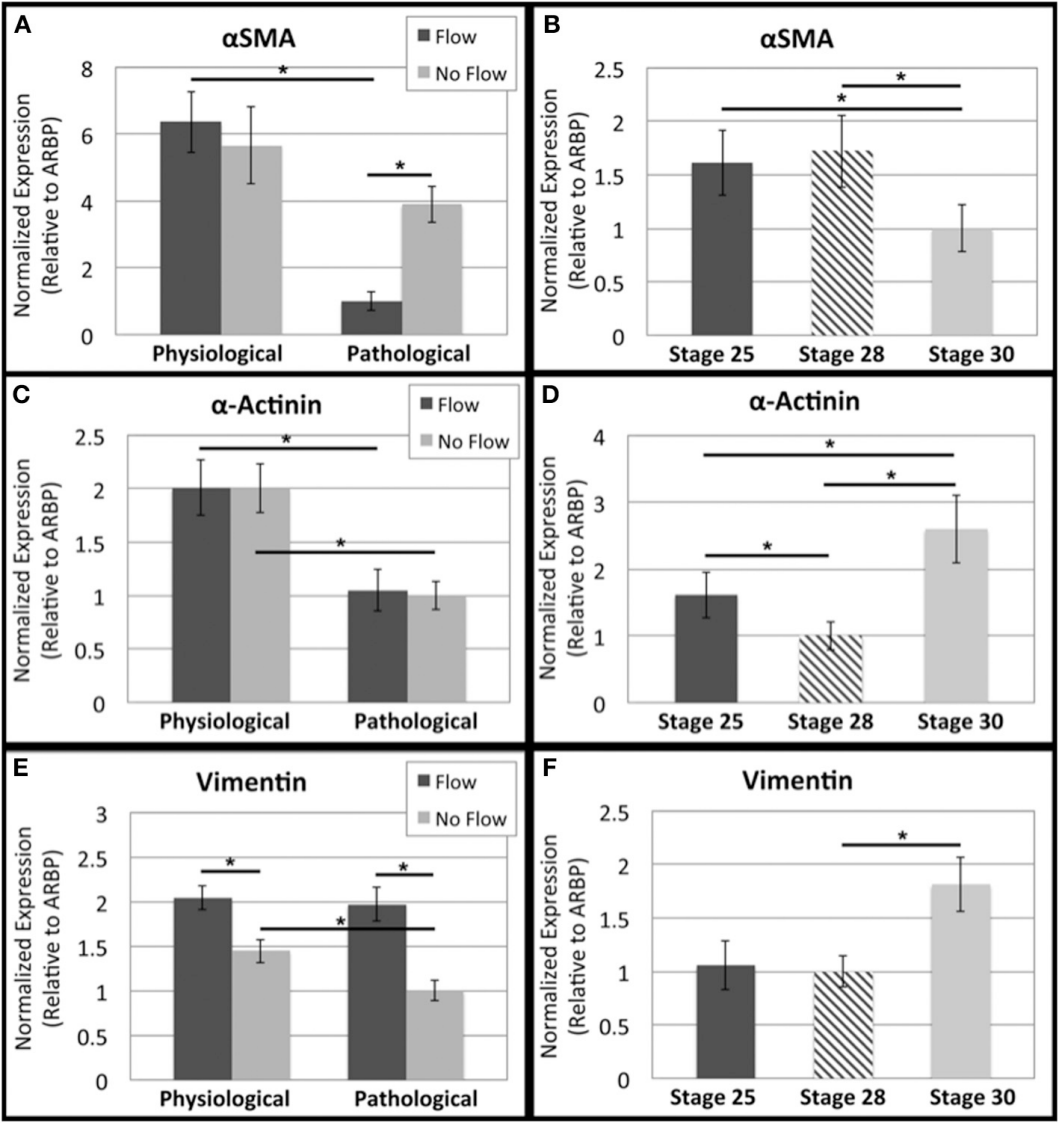
**Flow affects cytoskeleton remodeling transcript levels in outflow tract**. Quantitative Real Time PCR was performed on the four culture groups and on freshly dissected outflow cushions (OFCs) at three stages in development (HH stage 25, 28, and 30). **(A,B)** αSMA transcript level was highest in the presence of physiological flow and appears to decrease between HH stage 28 and 30. **(C,D)** α-Actinin transcript level was the same between flow groups and their controls, but transcript was greater in the physiological groups. Transcript appears decrease from HH stage 25 to 28 and increase from HH stage 28 to 30. **(E,F)** Vimentin transcript level was higher in the presence of flow and appears to increase from HH stage 28 to 30. (^*^P < 0.05).

Finally, to look further at possible cushion cell phenotype, αSMA, α-actinin, and vimentin transcripts were analyzed. αSMA and α-actinin flow group transcript levels both decreased with time (Figures 10A,C, respectively). Vimentin transcript levels were upregulated in response to flow with no significant change between the two flow groups (i.e. time cultured under flow) (Figure 10E). αSMA transcript decreased from HH stage 25 to 30, while α-actinin transcript increased from HH stage 25 to 30 (Figures 10B,D, respectively). An increase in vimentin transcript was seen from HH stage 28 to 30 (Figure 10F).

The normalized relative quantity (NRQ) ratios between different group combinations are listed in Table 3 (*in vitro* culture groups) and Table 4 (“*in vivo*” samples). In each table, a color gradient is applied to compare the relative magnitudes of each NRQ ratio. Bright red is indicative of a significant decrease (*P* < 0.05) while bright green is indicative of a significant increase (*P* < 0.05). White cells indicate little to no difference between the compared groups.

**Table 3:**
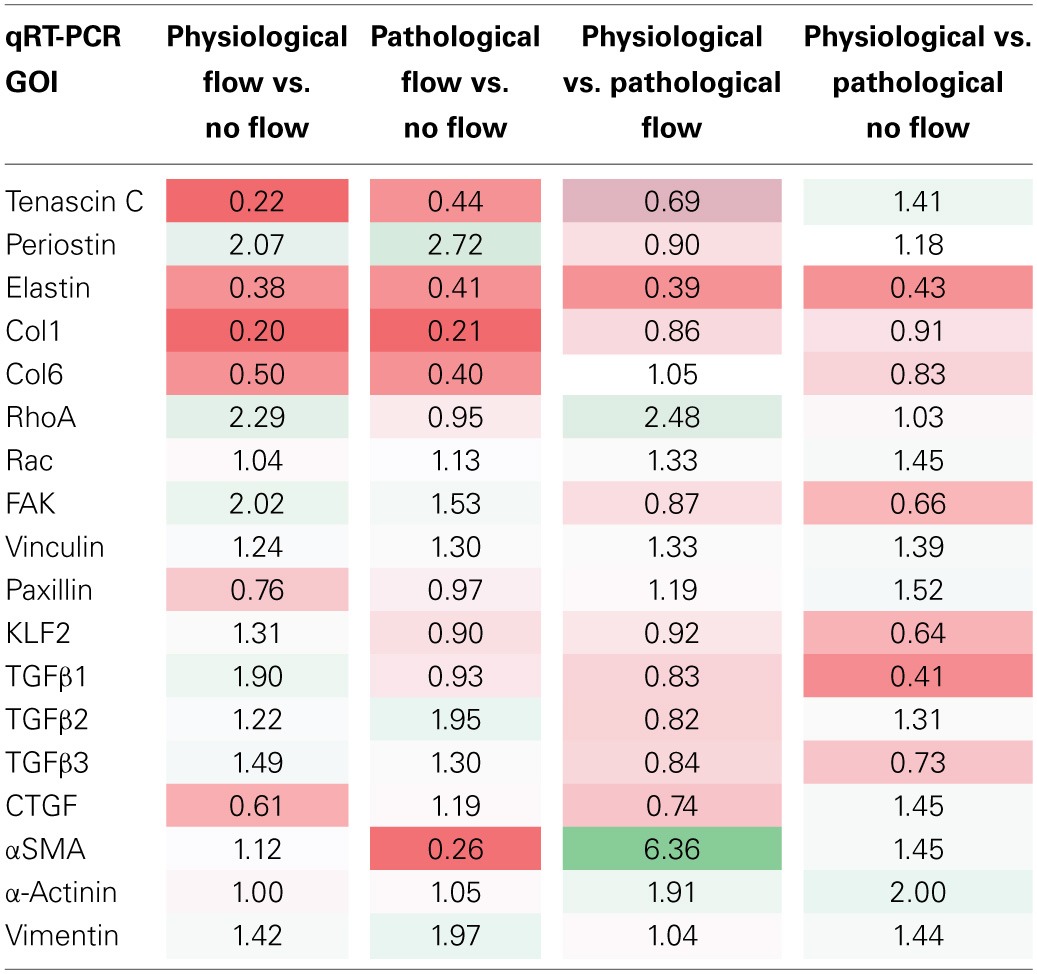
**Comparison of NRQ ratios in *in vitro* oft experiments**.

**Table 4:**
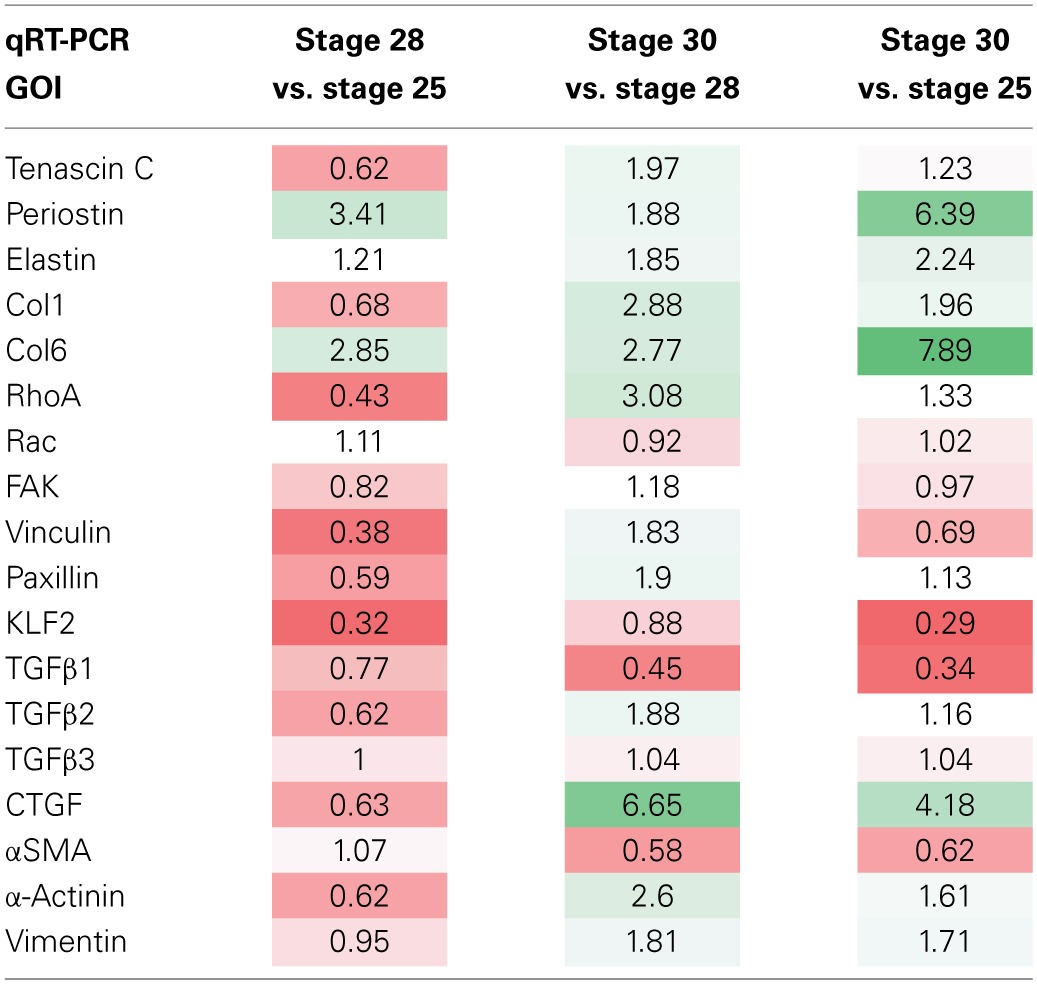
**Comparison of NRQ ratios in OFT for three OFT developmental stages**.

### Flow alters OFT cell death and proliferation

**Figure 11:**
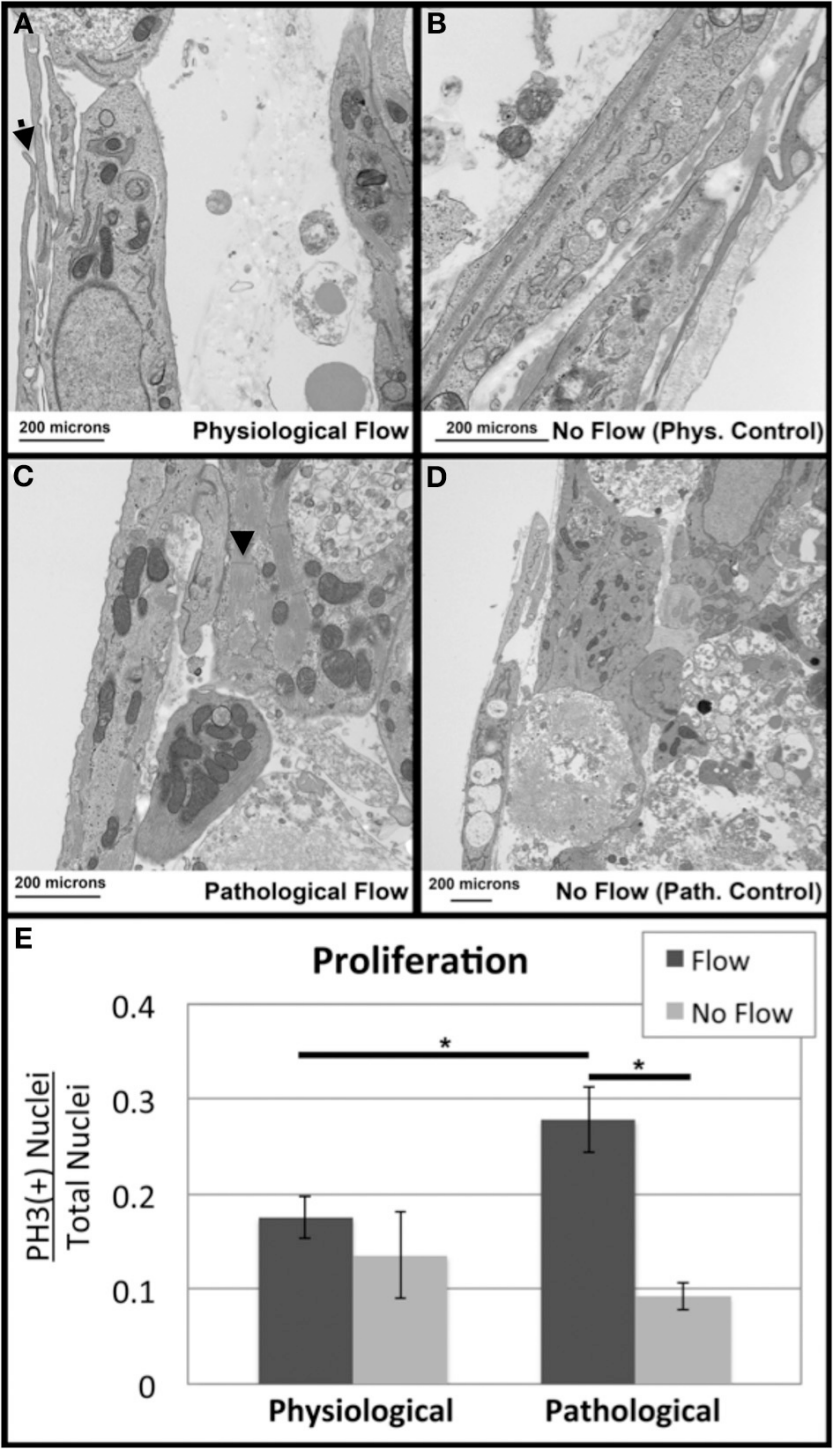
**Flow alters outflow tract explant cell death and proliferation**. Transmission Electron Microscopy was performed to investigate subcellular architecture. All samples contained myocyte cells as indicated by the presence of sarcomeres (arrowheads) and centrally located nuclei **(A)** Samples subjected to physiological flow exhibited an endothelial layer lining the flow side of the cushion, as well as ECM deposition and membranous protrusions (arrow). **(B)** Physiological no flow controls had some dead cell debris beneath the luminal cell layer. **(C)** Pathological flow groups did not show a luminal endothelial layer but showed MCs surrounded by ECM and cell debris. **(D)** Pathological no flow controls were similar to physiological no flow controls. Scale bars are all 200 μm. **(E)** Confocal microscopy was performed on sample groups for P-Histone H3 as a proliferative marker. P-Histone H3 (+) nuclei relative to total nuclei (Dapi) were quantified and indicated increase proliferation in the case of pathological flow. (^*^P < 0.05).

To look at the ultrastructure of the OFC culture groups, transmission electron microscopy (TEM) was carried out. Numerous sarcomeres with associated z-bands were observed (arrowheads Figure 11). These cells, which were present in all groups, also had centrally located nuclei indicating that they were cardiac myocytes (Figures 11A–D). In contrast, endothelial cells were only observed in the physiological flow and no flow control groups (Figure 11A,B, respectively). In physiological flow group, membranous protrusions were observed in on the luminal side of these endothelial cells (Figure 11A). However, no microtubules were associated with these protrusions in any of the groups (Figure 11A). A loose network of ECM was observed in both flow groups (Figures 11A,C). All groups showed evidence of cell debris; however, this was more wide spread in the supraphysiological, pathological flow group (Figure 11C).

Because p-Histone H3, is expressed in the myocardium during pathological tissue apoptosis (de Vlaming et al., 2012), sections from the four culture groups were stained with p-Histone H3 and Dapi and the number of positive nuclei vs. total nuclei per section was calculated. Pathologically high levels of flow (supraphysiological) were associated with increased cellular proliferation compared to the no flow control and the physiological flow group (Figure 11E).

## Discussion

The goal of the experiments presented in this study was to characterize OFC cellular response to predicted physiological or pathologically high (supraphysiological) flow-induced forces. This response was characterized as normal or abnormal based on tissue morphology, fibrous ECM protein expression, and localization, comparison of many different gene transcripts associated with the post-EMT developmental stage, and subcellular ultrastructure. Particular changes in all five of these areas are associated with valve diseases, the most common CHDs. As valve disease is problematic and costly to both children and adults almost equally (Schoen, 2008), understanding the mechanistic progression of valve pathogenesis is of extreme importance.

These experiments found that HH stage 25 OFT explants cultured under predicted physiological flow lead to the formation of a compact mound, similar to the cushions that are seen during early stages of development. Pathologically high flow cultures lead to thin leaflet formation with a reduced scaffold interface area. This “leaflet detachment” may represent failure of the tissue's cellular architecture under pathologically high loading conditions. In the absence of flow, a loose network of cells formed that occluded the lumen. Cells in the no flow controls invaded the walls of the collagen scaffold, indicative of a possible EMT re-initiation. This was also observed in our previous report using AV explants (Tan et al., 2012).

A interesting difference between these studies with OFT tissues and our previous AV cushion studies are that fibrous ECM gene transcripts were upregulated by flow in the AV cushions, but not in OFT cushions. However, fibrous ECM protein deposition, which increases during normal development, was found to increase in response to both levels of flow in OFT tissues. The no flow controls, by comparison, had sparse deposition of these proteins, which is, again similar to our AV cushion studies. This indicates that flow plays a central role in stimulating the fibrous ECM protein deposition the OFT post-transcriptionally. Collagen and tenascin C have been found to colocalize early on in OFC development (Tan et al., 2011), and in these experiments, Col1 and tenascin C colocalized only in response to flow. Elastin also increased in response to flow and accumulated throughout the OFT explant cultures, with higher concentration on the downstream side. Simulation results of flow through the molded collagen scaffold indicated downstream eddy formation (Figure 1B), so this elastin localization may be associated with localized stresses due to the eddies.

Previously, we found increased fibrous ECM protein expression due to extremely low levels of laminar flow past AV explants was attributed to increased tissue stiffness (Tan et al., 2012). While ECM stratification is extremely important to valve mechanics late in development, fibrous ECM proteins such as collagen are relatively sparse early in development and the cellular cytoskeleton becomes important in determining overall tissue mechanics. F-actin and fibronectin were found to localize throughout the explant tissue in the 4 culture groups, and exhibited higher staining concentration at the scaffold wall interface. Both F-actin and fibronectin expression were higher under physiological flow, and pathological flow appeared to reduce expression. This data indicates that physiological flow increases actin polymerization and thus may promote the mechanical contribution of the cytoskeleton, while pathologically high flow has the opposite effect.

Though flow increased deposition of fibrous ECM proteins in OFT explants, flow decreased tenascin C, elastin, Col1, and Col6 transcripts and increased the periostin transcript. The expression of fibrous ECM protein such as collagen is complex with numerous post-transcriptional regulatory mechanisms. Importantly in our experiments, flow was found to increase protein deposition in the OFT, which is critical in *in vivo* development. In our previous report using AV explants, these transcripts were all increased (Tan et al., 2012). This indicates that flow regulation of these transcript levels may be more complex in the OFT. Perhaps different waveforms are necessary to drive increases in transcript levels but the steady state flows delivered in our current system are capable of driving protein deposition.

Flow stimulated regulation of periostin appears to be different than the other fibrous ECM proteins investigated here. Periostin, which is necessary for collagen fibrilogenesis, is responsive to TGFβ3, a gene that exhibits increased transcript in response to flow (Norris et al., 2007, 2009; Hinton and Yutzey, 2011). Previous *in vitro* studies using AV explant cultures showed that periostin was not induced by the RhoA pathway, and it was proposed that regulation of periostin was regulated by other pathways (Tan et al., 2012). Periostin was; however, upregulated by LPA, so possibly, another Rho family GTPase, such as Rac or CDC 42, is involved in periostin regulation. All fibrous ECM protein transcripts were found to increase in OFTs from HH stage 28 to 30. Transcripts of the flow-responsive genes, KLF2, TGFβ123, and RhoA increased in response to physiological flow, while CTGF decreased in response to flow. This opposing trend is the same in the *in vivo* cases (i.e. if CTGF transcripts are upregulated, then KLF2, TGFβ123, and RhoA transcripts are downregulated). While RhoA/actin dynamics are necessary for CTGF and tenascin C induction (Asparuhova et al., 2009), they are also necessary for other cell processes such as cytoskeletal actin organization. Possibly, at this early stage in development, fibrous ECM proteins, while they are characteristically more organized, are not the primary cellular output in response to external mechanical stimuli.

Transcription of migration-associated molecules including FAK, RhoA, vinculin, and αSMA (a myofibroblast marker) increased when explants were exposed to physiological flow. However, when cultured under pathological conditions, RhoA activity significantly decreased and pathological flow decreased αSMA transcription. FAK is thought to regulate polarized RhoA and Rac activity during cell migration, so increases in migratory molecule transcription may indicate a healthy remodeling process in response to flow-induced loading (Li et al., 2005). Further, RhoA, Rac, and FAK transcripts do not change from HH stage 25 to 30, indicating that these cytoskeletal remodeling processes are ongoing and necessary throughout development. TEMs also showed some possible signs of cell migration in the physiological flow group, including membranous protrusions associated with cushion development (Markwald et al., 1975). Pathological levels of flow appeared to drive cell apoptosis, indicated by qualitative assessment of dead cell debris in TEMS, and quantitative increased proliferation, identified by an increase in p-Histone H3 positive cells, which are both characteristic of valve pathology (Pfaffl, 2001).

The finding of sarcomere containing cells in the TEM studies indicates that myocardialization of the OFT explants was in progress. Cardiac myocytes were found in all study groups, but appeared to be more abundant in the flow groups. Thus, flow may have a role in this step of OFT development. The endothelial cell protrusions found on the luminal surface of the physiological flow cultures provide a potential mechanism of flow sensing in these cells. We looked for primary cilia in these structures as they have been reported by others (Egorova et al., 2011). However, none were found in our studies. This may be due to the levels of flow used in this study, as these cilia have been reported to retract in regions of high flow. Clearly a more in depth study is required to clarify these questions.

The study presented here illuminates the importance of flow-induced loading on cushion developmental mechanisms that ultimately regulate healthy vs. pathological valve mechanics. Numerous CHDs are attributed to OFT malformation, involving both the valves and the arterial poles (Lucitti et al., 2005, 2006; Schoen, 2008; Freund et al., 2012). Using an *in vitro* system of OFT explant development, physiological levels of flow were shown to promote cellular processes characteristic of normal valve development, while supraphysiological levels of flow were shown to impede tissue development and drive pathobiology. The data presented here provides a necessary contribution to the growing field of heart valve engineering. By shedding light on the role of flow-induced forces on these important developmental processes, new possibilities can be laid out in the development of better therapies and replacements for both CHD and non-CHD attributed valve disease.

## Limitations of the study

The central goal of these studies was to determine the role that fluid flow plays in the fibrous development of the aorticopulmonary septal and valvular tissues. A significant limitation of our study is that we were only able to deliver steady flow in this system. *In vivo* flow dynamics are much more complicated during morphogenesis of the outflow tract. Vortical, pulsatile, and even regurgitant flows are present during development of the arterial pole of the heart. To better model these fluid dynamics, we are developing flow systems that can deliver these more complex flows to cardiovascular tissues as they are likely to influence morphogenesis.

Another limitation of this study is in the experimental design. We chose to model the development of stenosis after an initial “normal phase” of development. Thus, pathological studies lasted two days longer than the physiological studies. This means that the differences that we found between the supraphysiological and physiological conditions could be due to the lengthened culture time and not the increase in shear stress. We plan to use our more complex flow bioreactor system to parse out these questions and many others with greater resolution and precision.

## Funding

Funding for this work was provided by NIH grants HL086856 (Richard L. Goodwin) and DE019355-02 (Michael J. Yost).

### Conflict of interest statement

The authors declare that the research was conducted in the absence of any commercial or financial relationships that could be construed as a potential conflict of interest.
